# Viral Sepsis in Children

**DOI:** 10.3389/fped.2018.00252

**Published:** 2018-09-18

**Authors:** Neha Gupta, Robert Richter, Stephen Robert, Michele Kong

**Affiliations:** ^1^Division of Pediatric Critical Care Medicine, Department of Pediatrics, University of Alabama at Birmingham, Birmingham, AL, United States; ^2^Division of Pediatric Critical Care Medicine, Cedars-Sinai Medical Center, Los Angeles, CA, United States

**Keywords:** viral sepsis, sepsis, viral infections, viral coinfections, secondary bacterial infections, pediatric, children

## Abstract

Sepsis in children is typically presumed to be bacterial in origin until proven otherwise, but frequently bacterial cultures ultimately return negative. Although viruses may be important causative agents of culture-negative sepsis worldwide, the incidence, disease burden and mortality of viral-induced sepsis is poorly elucidated. Consideration of viral sepsis is critical as its recognition carries implications on appropriate use of antibacterial agents, infection control measures, and, in some cases, specific, time-sensitive antiviral therapies. This review outlines our current understanding of viral sepsis in children and addresses its epidemiology and pathophysiology, including pathogen-host interaction during active infection. Clinical manifestation, diagnostic testing, and management options unique to viral infections will be outlined.

## Introduction

Sepsis is a leading cause of pediatric mortality (1). Defined as systemic inflammatory response syndrome in the presence of a suspected or confirmed infection, it is a clinical syndrome principally characterized by dysregulation of the host innate immune response and may result in an immune phenotype of coexistent systemic inflammation and immunosuppression (2). Pathological cross-talk between inflammatory and coagulation cascades, complement activation, and neuroendocrine signals wreak havoc on homeostatic controls. This hyperinflammatory response has untoward effects on the cardiopulmonary system, vascular endothelium, and gut, precipitating progressive organ dysfunction until the host succumbs (3). The morbidity, mortality, and costs associated with pediatric sepsis impose a significant burden on the healthcare community and global economy (4, 5). Watson et al reported a mean hospital length of stay of 31 days, with approximately $2 billion spent annually in healthcare cost associated with severe pediatric sepsis (1). International guidelines for management of sepsis and septic shock stress the importance of rapid resuscitation, prompt antimicrobial administration, and supportive care of organ dysfunction as the mainstays of pediatric sepsis treatment (6).

Viral sepsis can be defined as a severe inflammatory response to a suspected or confirmed viral infection. However, making the definitive diagnosis of viral sepsis in a child is particularly challenging for clinicians. The astute clinician must incorporate the patient's history of present illness, physical exam, laboratory and radiographic data to determine the likelihood of a viral etiology for sepsis. Even with a positive viral test, limitations of the testing result should be considered. Despite these challenges, timely diagnosis of viral sepsis has significant implications on clinical management, including guiding the use of appropriate antiviral therapy and informing isolation and containment strategies. Moreover, timely diagnosis of viral sepsis may prevent unnecessarily prolonged antibacterial treatment exposure and thus could help prevent consequent antibacterial resistance and deleterious effects on the host microbiome. This review outlines our current understanding of viral sepsis in children, including its epidemiology and the pathophysiology of the viral-host response during active infection. The clinical manifestations, appropriate diagnostic testing, and management unique to viral infections are outlined.

### Epidemiology

The true incidence of viral sepsis, particularly in the pediatric population, remains unknown. Since bacterial sepsis is amenable to treatment and is presumably more common, viral testing is frequently foregone in the acute presentation of sepsis. However, a recent study of adult patients with sepsis showed that viral respiratory pathogens, namely influenza A virus, human metapneumovirus, coronavirus, and respiratory syncytial virus (RSV), were overlooked in 70% of patients (7). In a multi-national epidemiological study of children with severe sepsis, an infectious etiology was only proven in 65% of patients and out of these, approximately one-third had a viral infection (8). The most frequent sites of infection were the respiratory tract (40%) and bloodstream (20%), with rhinovirus, RSV, and adenovirus most commonly isolated. In contrast, the Australia and New Zealand sepsis study group identified a pathogen in approximately 50% of patients with sepsis and septic shock (9). Of these patients, only one-fifth had a viral etiology, with RSV, cytomegalovirus (CMV), Epstein-Barr virus (EBV), herpes simplex virus (HSV), varicella zoster virus (VZV) and influenza being the most common viruses identified in this study. Recently, Ames et al. reported that 16% of pediatric patients who presented with septic shock had a primary viral disease (10). In another study of neonates with sepsis, bacterial etiology was found in only approximately 15% of cases, making viral infection more likely as a plausible cause of sepsis in these patients (11).

In the pediatric intensive care unit (PICU), influenza virus is a leading cause of viral sepsis and caries an especially high mortality rate (12). RSV has also been found to cause severe bronchiolitis and may present with sepsis, especially in children with history of premature birth, chronic lung disease, congenital heart disease or primary immunodeficiency (13, 14). Sepsis has also been observed in neonates with HSV, human parechovirus (HPeV) and enteroviral infection (15–18). Patients with immunodeficiency due to human immunodeficiency virus (HIV) infection are highly susceptible to viral sepsis depending on the stage of disease and access and response to the treatment (19). In these patients, common viral infections observed to cause sepsis include RSV, influenza, parainfluenza, adenovirus, CMV, EBV, and VZV (19). Diarrheal diseases secondary to viral infections can also lead to sepsis, especially in developing countries (20). Although rare, rotavirus has been associated with sepsis due to bacterial coinfection (21). Despite several large studies on viral sepsis in general (ref as above), as well as on specific viruses, in the absence of routine viral testing during the diagnostic evaluation of sepsis, the true incidence of viral infection as the cause of sepsis remains unclear.

### Pathophysiology and host response to viral sepsis

The host response to infection consists of a multitude of simultaneous processes designed to neutralize the infectious threat and initiate repair of injured tissue. Sepsis is characterized by systemic and dysregulated inflammation, which can lead to a vicious cycle of vascular endotheliopathy, microcirculatory hypoperfusion, intestinal barrier dysfunction, circulatory shock, mitochondrial failure, and death (22–24). Moreover, the concomitant compensatory anti-inflammatory response syndrome that is characterized by lymphocyte apoptosis and immune paralysis predisposes the host to secondary nosocomial infection and latent viral activation (25, 26). The type of mechanisms employed vary by virus but generally result in some combination of (1) cytokine release, (2) endotheliopathy, and (3) host cytotoxicity (27). While an in-depth review of the pathogenesis of all human disease-causing viruses is beyond the scope of this manuscript, we have outlined the general pathophysiology below, highlighting major illustrative viral examples where possible.

#### Cytokine release (Figure 1)

Pathogen-recognition receptors (PRRs) are cellular sensors that recognize specific molecular structure of a pathogen (28). Toll-like receptors (TLRs) and retinoic acid-inducible gene-I (RIG-I)-like receptors (RLRs) are two types of PRRs that are involved in viral sensing (28). TLRs, which are found on the cell surface or within endosomes of monocytes, macrophages, dendritic, epithelial and endothelial cells, encounter pathogen-associated molecular patterns (PAMPs) (29, 30). Intracellular TLR-7 and TLR-8 recognize single-stranded Ribonucleic Acid (RNA) of viruses like HPeV, the enteroviruses, human metapneumovirus, and influenza; intracellular TLR-9 recognizes double-stranded (ds) DNA of viruses like the herpes viruses (e.g., HSV-1 and -2, EBV), adenovirus, and CMV; and TLR-3 recognizes dsRNA produced during intracellular viral replication (31). TLR activation culminates in myeloid differentiation primary response 88 (MyD88, through TLR-7,-8, and -9) or Toll/Interleukin (IL)-1 receptor domain-containing adapter protein inducing IFN-β (TRIF, through TLR-3) activation (32). These proteins, in turn, activate nuclear factor kappa-light-chain-enhancer of activated B cells (NF-κB) and IFN-regulatory factor (IFR)-mediated cytokine transcription (33–35). RLRs are cytosolic innate immunity sensors for viral RNA. Three members of RLRs have been identified: RIG-I, melanoma differentiation associated factor 5 (MDA5], and laboratory of genetics and physiology 2 (LGP2) (36). RIG-I and MDA5 recognize dsRNAs in response to different RNA viruses and signal the production of pro-inflammatory cytokines and type-1 IFNs (37). Cytokine proliferation instigates a pro-inflammatory cascade that results in complement activation, neutrophil chemotaxis, cytotoxic cluster of differentiation (CD) 8+ T-cell recruitment, and protease release from leukocytes and endothelial cells, particularly trypsin (38) and heparanase (39). Trypsin is upregulated and released by the vascular endothelium (38) and has been shown to cleave circulating pro-matrix metalloproteinase (pro-MMP) released from macrophages to form activated MMPs (40). MMPs, in conjunction with heparanase, degrade the endothelial glycocalyx (41, 42). Moreover, viral particles induce reactive oxygen species generation by circulating neutrophils, eosinophils, and macrophages (43, 44) that further injure the endothelial glycocalyx (45) and activate NF-κB cell-signaling (46), propagating a positive-feedback loop that results in endotheliopathy and end-organ damage.

**Figure 1:**
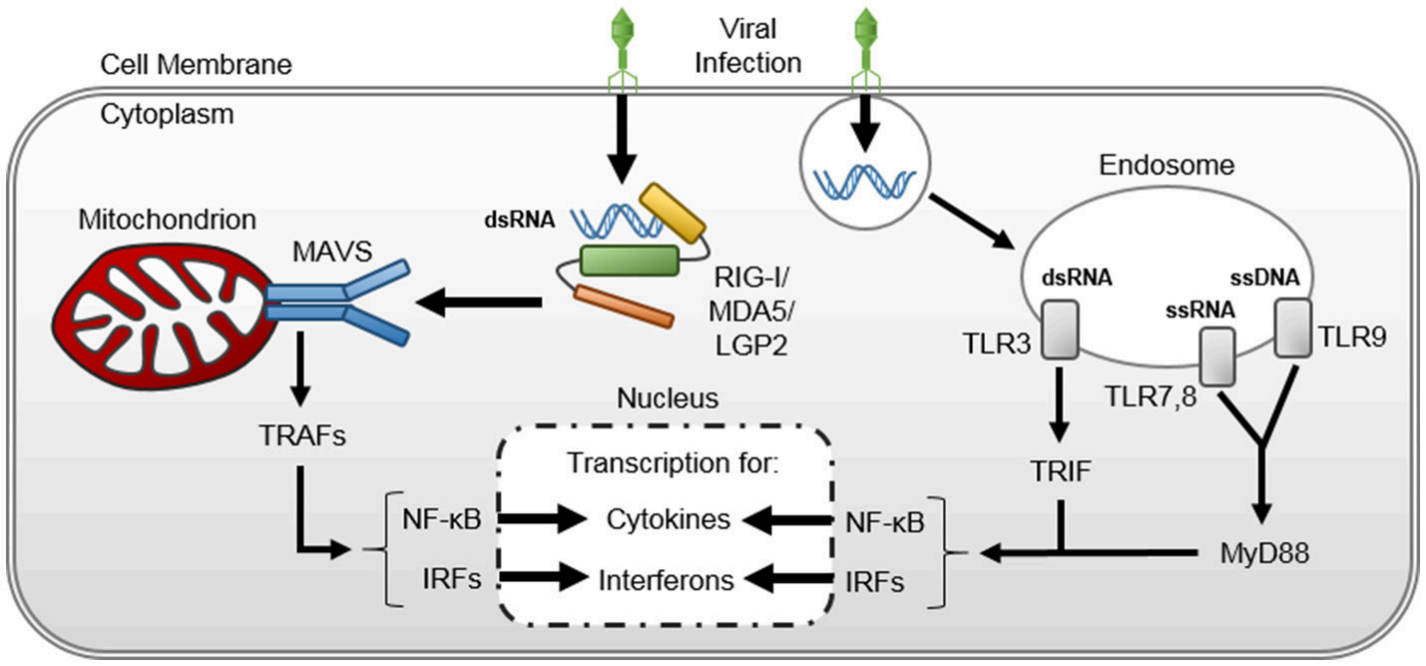
Viral-induced cytokine upregulation and release. Double stranded viral ribonucleic acids are (dsRNA) recognized within the host cellular cytosol by retinoic acid-inducible gene-I (RIG-I)-like receptors (RLRs)- RIG-I, MDA5, and LGP2. The RLRs bound to viral dsRNA undergo conformational change and complex with mitochondrial antiviral signaling (MAVS) protein on the mitochondrion surface. The RLR-MAVS interaction instigates an assembly of host proteins to activate TNF- receptor-associated factors (TRAFs), thereby inducing nuclear factor kappa-light-chain-enhancer of activated B cells (NF-κB) and interferon regulator factor (IRF)-mediated cytokine transcription in the host cell nucleus. Viral nucleic acids are also recognized within Toll-like receptors (TLRs) within host cell endosomes, triggering myeloid differentiation primary response 88 (MyD88) and Toll/interleukin-1 receptor-domain-containing adaptor-inducing interferon-β (TRIF) pathways that also activate NF-κB and IRF-mediated cytokine transcription in the host cell nucleus.

#### Endotheliopathy (Figure 2)

Systemic viral dissemination appears to be the etiology of viral sepsis. The exact mechanisms by which viruses that are normally isolated to the respiratory or integumentary epithelium reach the bloodstream are not known. However, it is plausible that viremia occurs through direct invasion of epithelial cells (or neurons as in case of HSV or varicella disease) to reach the surrounding vasculature (47). Once in the blood, the virus may induce endothelial glycocalyx degradation by activating leukocytes, platelets, and endothelial cells to secrete MMPs and heparanase that target glycocalyx components (39, 48). Endothelial glycocalyx disruption exposes selectins and intracellular adhesion molecules, making them available for leukocyte adhesion and activation (49). Glycocalyx degradation also releases heparan sulfate that may bind and activate antithrombin III and exposes membrane-bound glycoprotein Ib/IX/V complexes (50) that can bind circulating von Willebrand factor (51) and P-selectins on platelets (52, 53), precipitating coagulopathy. Moreover, loss of integrity of the protein-rich glycocalyx alters the microvascular fluid equilibrium between the vascular lumen and subglycocalyx, increasing fluid and macromolecule filtration through the endothelium to the surrounding interstitium (54). The end result of endothelial glycocalyx damage is pro-inflammatory propagation and vascular leak that can compromise organ function.

**Figure 2:**
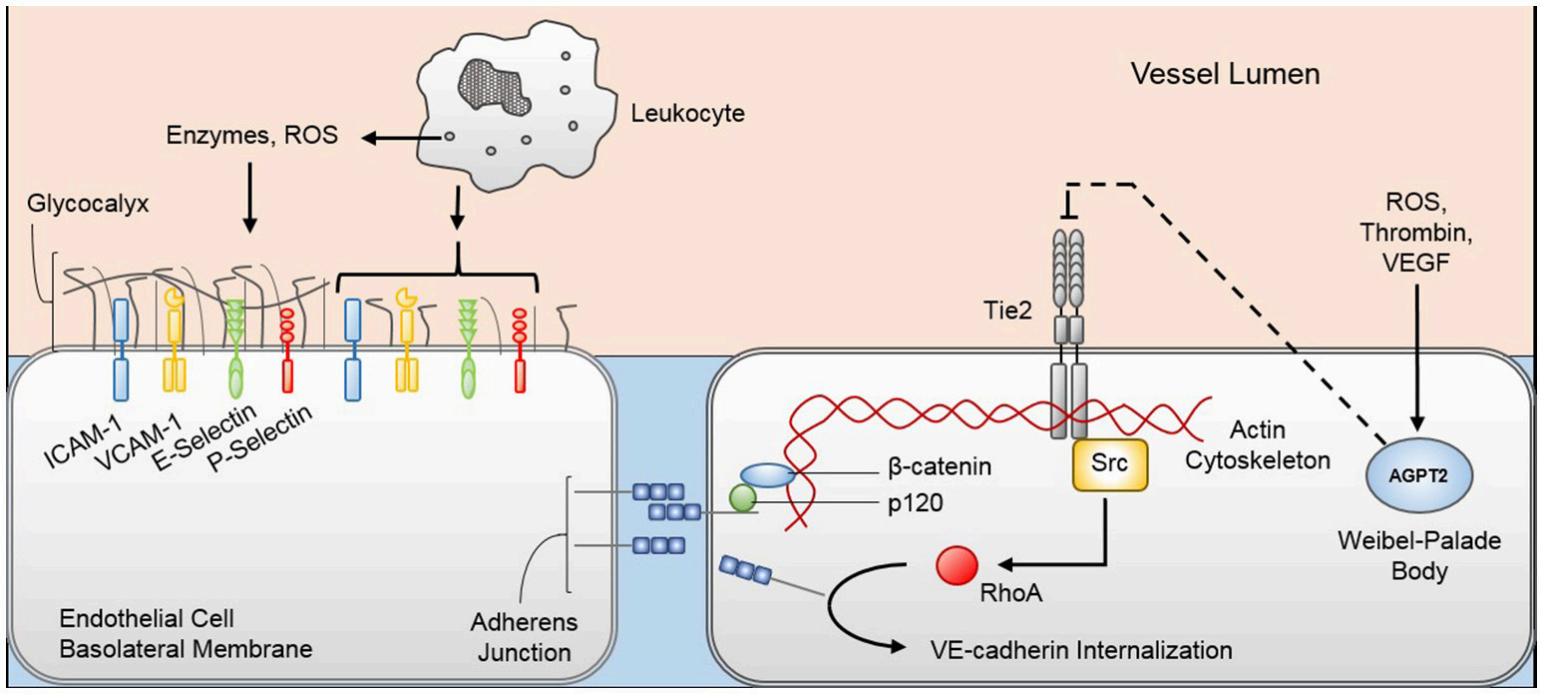
Pathophysiologic mechanisms of viral-induced endotheliopathy. Innate immune system activation during viral sepsis precipitates leukocyte degranulation and release of enzymes and reactive oxygen species (ROS) that degrade the endothelial glycocalyx. Denuded endothelial glycocalyx exposes cellular adhesion molecules (e.g., ICAM-1, VCAM-1, E-selectin, P-selectin) that increase the margination and activation of leukocytes, further promoting the inflammatory response. Additionally, inflammatory mediators, namely thrombin, ROS, and vascular endothelial growth factor (VEGF), promote Weibel-Palade body exocytosis, releasing angiopoietin-2 (Agpt-2) into the circulation. Agpt-2 antagonizes the endothelial cell Tie2 receptor, allowing the Src-mediated RhoA enzyme to reconfigure the endothelial cell cytoskeleton and promote VE-cadherin internalization from the adherens junction. Loss of glycocalyx and adherens junction integrity permits increased trans-cellular protein and fluid movement from the vascular lumen to the interstitium. ICAM-1, intercellular adhesion molecule 1; VCAM-1, vascular cell adhesion molecule 1.

Circulating viral particles may also induce endothelial cell structural changes that lead to barrier disruption and further vascular leak. Endothelial cells are anchored to each other through adherens junctions comprised predominantly of vascular endothelial (VE)-cadherin, which is attached to the endothelial cytoskeleton through beta-catenin and p120-catenin (55, 56). In human endothelial cells, pathogenic strains of hantavirus appear to bind to cellular surface β3-integrins, thereby promoting VE-cadherin internalization and adherens junction destabilization (57). VE-cadherin destabilization may also be mediated through the cellular membrane Tie2 receptor. Tie2 receptor is activated by angiopoietin-1 (Agpt-1) that is derived by periendothelial cells (58, 59). When activated, the Tie2 receptor activates PI3K/Akt cell-survival signaling and Rac1-mediated cytoskeletal stabilization (60). Inflammatory mediators, such as thrombin (61), reactive oxygen species (62), and VEGF (63), stimulate endothelial cell Weibel-Palade body exocytosis, releasing the Tie2 antagonist Agpt-2 (64). Agpt-2 acts in an autocrine fashion to inhibit Tie2 signaling, thereby promoting RhoA kinase activity and VE-cadherin destabilization (60). Mice infected with a pathogenic strain of H3N2 influenza virus develop acute lung injury that is rescued by the Tie2 agonist vasculotide (65), suggesting that the Tie2 receptor is integral in the development of endotheliopathy during viral sepsis. The exact mechanisms each virus employs to induce endothelial cell dysfunction are not clear; however, the typical presentation of capillary leak with viral sepsis suggests a common pathway by which endothelial integrity is compromised.

#### Host cytotoxicity (Figure 3)

Viral-induced host cytotoxicity is mediated by cytopathic effects, cellular reprogramming, and/or initiation of host immune cytotoxic responses. Viruses take over and utilize host intracellular machinery to replicate, depleting host cells of energy stores and transcription potential. Furthermore, viral-infected cells may be activated for caspase-dependent apoptosis (66). Viruses may also indirectly promote apoptosis through macrophage reprogramming. Macrophages infected with H5N1 avian influenza have been shown to upregulate production and release of tumor necrosis factor (TNF)-related apoptosis-inducing ligand (TRAIL) that promote T-cell apoptosis (67). Though the effect of TRAIL on other cell lineages was not determined, it is plausible that the effect seen in T-cells may be more diffuse. Systemic viral dissemination from sites of primary infection (e.g., human metapneumovirus in the respiratory tract or HPeV in the gastrointestinal tract) may occur through these apoptotic mechanisms, whereby new virions are released, infect local endothelial cells, and cause further cellular apoptosis and systemic viral spread. The invasiveness and pervasiveness of the viral infection is likely dictated by cell tropism and genetically determined virulence as viruses with greater cytopathogenicity, such as H5N1 avian influenza (66) and HPeV-3 (68), are more likely to cause sepsis in immunocompetent hosts than viruses with typically minimal associated cytopathology (e.g., RSV or parainfluenza) (69). Lastly, effectors of the host immune system, such as natural killer (NK), cytotoxic CD8+ T cells and complement, attack, and destroy virally infected host cells.

**Figure 3:**
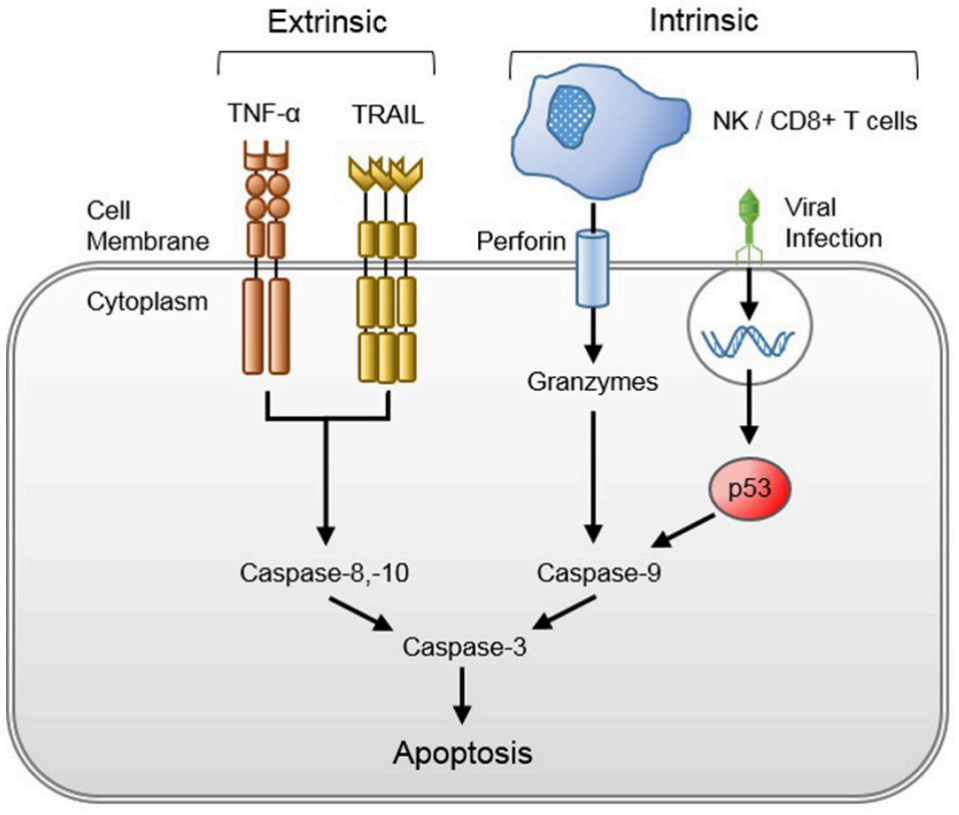
Viral-induced host cytotoxicity through the extrinsic and intrinsic apoptotic pathways. Tumor necrosis factor-alpha (TNF-α) is released after viral recognition by the innate immune system, and TNF-related apoptosis-inducing ligand (TRAIL) is released from viral-reprogrammed macrophages, both of which bind their respective host cell surface death receptors to activate the extrinsic apoptotic pathway. Natural killer (NK) and cluster of differentiation 8 (CD8+) T cells inject perforin into the membranes of infected host cells, through which they secrete granzymes that elicit the intrinsic apoptotic pathway. The intrinsic pathway may be activated after viral protein recognition by the host cell or suicide gene insertion by cytotoxic viruses.

Viral pathogenesis in children also varies according to the degree of host immunocompetence. Generally, young infants have significantly reduced TLR expression, antigen-presenting cell activity, NK cell responsiveness, T-cell functionality, B-cell maturity, and complement concentration (70). This immaturity of the developing immune system places young infants at significantly higher risk for severe disease from viruses that would typically cause minimal harm to older children and adults (e.g., HSV, HPeV, enteroviruses, CMV). Similarly, children with congenital or acquired immunodeficiencies are more susceptible to viral pathogens. Specific immunodeficiencies that place children at higher risk for viral sepsis include NK cell deficiency, interferon (IFN)-γ receptor deficiency, TLR-3 deficiency, nuclear factor-kappa B essential modulator deficiency, severe combined immunodeficiency, severe T-cell lymphopenia in DiGeorge syndrome, agammaglobulinemia, and hyperIgM syndrome (71). Severe RNA viral infections have also been observed in patients with loss-of-function mutation of the IFN induced with helicase C domain 1 (IFIH1) gene that encodes the RLR MDA5 (36, 37, 72). Moreover, children receiving immunomodulatory or immunosuppressive therapies due to malignancy, transplantation, or autoimmune disease are more susceptible to viral infection or reactivation.

### Clinical features and risk factors for viral sepsis

The constitutional symptoms and clinical features of viral sepsis are frequently indistinguishable from bacterial or fungal sepsis. Presenting symptoms and signs include fever, chills, rash, respiratory distress, nausea, vomiting, diarrhea, dysuria, confusion, and altered mental status. None of these symptoms is pathognomonic of sepsis, let alone viral induced sepsis. Moreover, classic features of systemic inflammation might not be seen in every individual, especially in immunocompromised children. Fever is one of the most common symptoms seen in septic children, attributable to the pyrogenic activity of IL-1, IL-6, IFNs, and TNF-α. It has been observed that these substances increase prostaglandin E2 synthesis in the hypothalamus (73, 74), resulting in the elevation in the host central nervous system core temperature set-point regulated by the pre-optic and dorsomedial hypothalamic nuclei (75). Hypothermia, on the other hand, is a less frequent but more specific indicator of sepsis that may be predictive of illness severity and death, especially in younger children and chronically debilitated patients (74). Injury to the vascular endothelium may result in broad array of failing organs that manifests as confusion, nausea, vomiting, diarrhea, oliguria, and coagulopathy. A myriad of cardiopulmonary manifestations ranging from mild tachypnea and tachycardia to acute respiratory distress syndrome and shock can be seen (76). The presenting symptoms usually depend on the type of virus. Clinical presentation in patients with respiratory viral infections can range from completely asymptomatic to severe respiratory distress due to pneumonia. Diarrheal illness has been observed in patients infected with rotavirus, norovirus, enterovirus, and adenovirus. VZV and HSV infection may present with vesicular rash. Children with HSV or arbovirus infection may have confusion, altered mental status or seizures from encephalitis. Elevated transaminases are common with HSV and enteroviral infections which may be complicated by hepatitis, coagulopathy and encephalitis. Neonatal HPeV infection can mimic other enteroviral infections in the initial presentation. Often these patients present with fever, rash, irritability, feeding intolerance, and seizures (17). They can develop sepsis like illness and encephalitis. Patients with acute HIV infection often have flu-like symptoms such as fever, headache and rash, which usually resolve spontaneously. These patients soon enter a phase of clinical latency until they develop acquired immunodeficiency syndrome, usually heralded by acquisition of an opportunistic infection.

As with other types of sepsis, virus-induced sepsis requires a high index of suspicion, especially in very young children and those with chronic medical conditions. Neonates and young infants are at higher risk of sepsis from HSV, HPeVs, and enterovirus. HSV is usually acquired perinatally from mothers with genital herpes. Mothers with primary herpes are more likely to transmit the infection when compared to those with recurrent and non-primary herpes (77). Nielsen et al reported that second born children are at higher risk of HPeV-3 infection than the firstborn (78). Seizures, drowsiness and lethargy, and absence of oral lesions are associated with severe enteroviral infection in children (79). In RSV infection, comorbid conditions reported to increase the risk for severe infection include the history of prematurity, congenital heart disease, chronic lung disease, and immunodeficiency (13). In a recent study, Eggleston et al found that patients with metapneumovirus infection were more likely to be older and have congenital heart disease compared to RSV infected patients (80). In contrast, asthmatics and premature infants were at higher risk for rhinovirus infection (81). Finally, predisposing conditions for severe pediatric influenza infection include age less than 2 years; asthma; cardiac, renal, hepatic, hematologic, neurologic or neuromuscular conditions; long-term aspirin therapy; immunosuppressive therapy and residence in a chronic care facility (82). Risk of mother-to-child perinatal HIV transmission is higher in mothers with CD4 count < 200 cells/μL and lower in infants receiving antiretroviral prophylaxis (83, 84). If patients with any of these conditions present with sepsis, diagnostic viral testing and appropriate empiric antiviral treatment should be strongly considered according to the individual's risk factors.

### Association with secondary bacterial infections and viral coinfections

Secondary bacterial infections are commonly associated with respiratory viral infections (85). In the winter of 1995–96, an outbreak of *Streptococcus pneumoniae* pneumonia developed in otherwise healthy children who had a preceding influenza A viral illness (86). During the 2009–10 influenza A pandemic, one third of critically ill children afflicted with influenza were diagnosed with concurrent bacterial infections (87). In this study, the leading three bacterial coinfections were *Staphylococcus aureus, Pseudomonas spp*., and *Haemophilus influenza* (87). In children hospitalized for RSV, *Haemophilus influenzae* and S*treptococcus pneumoniae* were the most common organisms isolated in those who developed bacteremia (88). These secondary bacterial infections may exacerbate innate immune dysfunction (89) and convey substantially increased risk of worse outcomes (90, 91). However, to date, the mechanisms underlying bacterial synergism and increased susceptibility to secondary bacterial infection in the setting of a preceding respiratory viral infection remain unclear. In general, this phenomenon appears to involve impairment of respiratory epithelial and innate immune system defenses. Viral destruction of the airway epithelium affects mucociliary clearance, allowing bacterial attachment to mucins and eventual colonization of the respiratory tract (92, 93). Additionally, viral-induced upregulation of IFN-γ and TNF-α may lead to a dysregulated host T-cell response, decreased neutrophil chemotaxis, and impaired macrophage phagocytosis that increases the host susceptibility to secondary bacterial pathogens (94). Upregulation of the surface platelet-activating factor receptor on epithelial cells and leukocytes by pro-inflammatory cytokines may also increase adhesion and invasion of certain virulent pneumococcal strains (95).

Rotavirus infection has also been associated with secondary bacterial infections (21). Although, the exact mechanisms leading to sepsis and organ dysfunction are unknown, a leading hypothesis entails translocation of bacteria and endotoxin through damaged intestinal epithelium into the splanchnic circulation, systemically increasing production of nitric oxide and circulating pro-inflammatory cytokines like TNF and IL-1β, and high mobility group box 1 protein, resulting in sequential organ failure (96). HIV infection can lead to apoptosis of CD4 T-lymphocytes, defective T and B lymphocyte function, decreased production of IFN-γ, IL-2 and immunoglobulins, and decreased NK cell activity (97–99). This leads to not only increased risk of secondary bacterial infections but also increased susceptibility to other viruses and intracellular organisms such as mycobacteria and *Pneumocystis jiroveci*.

Similar to bacterial coinfections, critically ill children can be simultaneously infected by multiple viruses. The course of illness in patients with viral coinfections depend on virus-virus interaction. Various mechanisms for disease virulence in viral coinfections have been proposed, including viral gene interactions, immunologic interactions and alteration in host environment (100). Even though the clinical significance such interactions is unknown, a study by Rhedin et al. reported increased risk of severe respiratory disease in patients with viral coinfections compared to those with single viral infections (101). Approximately 20% of the patients had viral coinfection and RSV, bocavirus and adenovirus were the most common viruses associated with coinfections (101). In another study performed in Canada on patients with respiratory viral infections, approximately 17% of the patients had viral coinfections (102). There was no difference in the risk of hospitalization or the severity of illness in patients with single viral infections and those with viral coinfections (102). Another study done in Greece revealed a much higher viral coinfection rate (42%) with most common coinfections with RSV, influenza, rhinovirus and parainfluenza viruses (103). Increased risk of hospitalization has been observed in patients with viral coinfections (103). However, systematic reviews and meta-analyses of children with viral coinfections have not shown any association with increased clinical severity (104, 105). Patients infected with HIV are at high risk of secondary viral infections such as CMV, HSV and respiratory viruses like RSV, influenza and metapneumovirus.

### Diagnostic testing

The diagnosis of viral sepsis is typically one of exclusion. Bacterial sepsis, whether primary or secondary, is usually of higher initial concern because failure to recognize this diagnosis and promptly administer systemic antibiotics has lethal consequences. Unfortunately, in our current state of limited antiviral therapies, even the prompt recognition and treatment of viral sepsis may not quickly improve a patient's clinical course. Nonetheless, early, definitive diagnosis of a primary viral septic process may inform treatment decision-making and help limit unnecessary systemic antibiotic administration. In symptomatic critically ill children, identification of a viral etiology can play an important role in the management and impact the outcome of these patients.

There are currently no standard approaches to viral diagnostic testing. Point-of-care (POC) antigen-based testing is relatively inexpensive and provides rapid detection of common respiratory viruses from a nasopharyngeal swab, such as RSV or common strains of human influenza. However, POC testing may lack the sensitivity needed to determine the etiology of life-threatening sepsis (106). Direct fluorescent antibody (DFA) testing may provide better specificity and a broader range of viral strain detection than POC testing, but the test depends on the collection of sufficient numbers of epithelial cells for adequate viral detection (107). Cell culture is the traditional gold-standard for viral diagnoses, including for HSV, however the long turn-around time for results significantly limits its utility for expedient diagnosis (108). Commercial or laboratory-developed nucleic acid amplification tests (NAATs) (e.g., polymerase chain reaction, PCR, or reverse transcription-loop-mediated isothermal amplification) may provide greater sensitivity and specificity than POC or DFA testing but requires sophisticated equipment and specially trained laboratory staff to complete (109, 110). NAATs have the added benefit of being highly multiplexed with new commercially available technology like Biofire® FilmArray® multiplex PCR (111). Unfortunately, the use of NAATs is limited by the high cost, delay in results and the inability to distinguish between viral nucleic acids from live viruses (112). Ultimately, the methods available for timely viral detection are limited by technique of sample collection and institutional resource availability.

Several limitations to the current diagnostic testing for causative viruses are worth noting, and results of viral testing always need to be interpreted with caution. For instance, although a type of enterovirus, HPeV cannot be detected on routine enterovirus PCR assay. HPeV-specific PCR is required to detect this virus in respiratory, CSF and stool samples of infected children and should be considered as a part of workup for neonates and young children presenting with sepsis (113). The clinical utility of viral respiratory PCR panels is also limited by their high rates of positive detections without clinical correlates. Detection of a virus in a patient with sepsis does not necessarily indicate causation. Some studies have shown that a respiratory virus can be detected in about one third of asymptomatic children (114, 115). Viral PCR testing is particularly difficult to interpret due to its high sensitivity for viral nucleic acids, making it challenging for the clinician to distinguish between active viral disease and viral nucleic acid or live viral carriage (112, 116, 117). A positive test could be a result of asymptomatic colonization, prolonged viral shedding or viral coinfection. In a study by Rhedin et al. comparing PCR results between symptomatic and asymptomatic patients, RSV, metapneumovirus and parainfluenza viruses had a significantly higher detection rate in children with acute respiratory infection, suggesting causation; however, other viruses (enterovirus, coronavirus, bocavirus, rhinovirus, and adenovirus) had an equally high detection rates in asymptomatic children (101). Because of these positive viral detections in asymptomatic children, it is important that clinicians consider the big picture, and factor in other pertinent information that may indicate an active ongoing bacterial infection before discontinuing antibacterial agents based on a viral assay. The use of serum biomarkers (see section below) and whole blood gene expression analysis (118) may serve a crucial role in this setting to discern viral from bacterial sepsis. When faced with a septic child who has an unusual sepsis presentation, does not respond to usual therapies, or has persistently negative diagnostic evaluations, a viral etiology must be considered and consultation with an infectious disease expert is recommended.

### Biomarkers for viral sepsis

Several serum biomarkers, including lactate, C-reactive protein (CRP), and procalcitonin (PCT), are used to guide management in sepsis and are an important part of early goal directed therapy (119). PCT is more commonly used in the ICU setting for early determination of the likelihood of a bacterial etiology for sepsis and to guide antimicrobial duration (120, 121). Serum PCT has been shown to be elevated in bacterial infections and is superior to CRP in assessing the severity and course of the disease (122). However, the mean sensitivity of PCT as a biomarker of sepsis remains low at 77%, with a specificity of 79% (123, 124). Unfortunately, in general, these biomarkers are non-specific in distinguishing bacterial vs. viral infection (125).

In recent years, several viral-specific biomarkers have been identified. Many transcriptional signatures have been designed to distinguish viral infections from bacterial infections as well as non-infectious conditions (126, 127). Zaas et al. identified a 30-gene signature to discriminate symptomatic influenza A-infected subjects from both healthy and bacterially-infected subjects (128). In a recent study by Herberg et al., a 2-transcript RNA signature [*FAM89A* and *IFI44L*) showed promising results in its ability to distinguish between bacterial and viral infections, demonstrating that the expression of *IFI44L* was increased in patients with viral infection, whereas expression of *FAM89A* was increased in patients with bacterial infection (118). Another recent study identified a four-gene expression signature in whole blood to distinguish viral infections from other etiologies (129). Human myxovirus resistance protein 1 (MxA) is an important intermediate product in the IFN-mediated antiviral response against a variety of viruses. Serum MxA levels are significantly higher in patients with viral infections compared to bacterial infections in pediatric population and thus may be an additionally useful biomarker to discriminate viral from bacterial illness (130).

### Preventive strategies and management

There is a paucity of data regarding treatment and management of viral infection. Supportive care is the current mainstay of therapy for most viral infections, particularly for respiratory viruses. Though broad-spectrum antibiotic therapy may be prudent until a bacterial source for sepsis has been definitively ruled-out, sustained antibiotic treatment has no role in the management of viral sepsis except in the case of bacterial coinfections. Many viral infections can be prevented with the use of hand hygiene, environmental decontamination, use of personal protective equipment, elimination of second-hand smoke, and isolation of infected children (131). Additional protection can be conferred by administering vaccines for common communicable viruses. These preventive strategies are of particular importance in high-risk patients. As the scope of available vaccines and anti-viral therapies remains rather limited, development of novel vaccines and treatment is critical (131).

For RSV infection, management is currently limited to passive immunization for at-risk infants. Palivizumab, an RSV-specific monoclonal antibody, is Food and Drug Administration (FDA) approved for the prevention of infection in high-risk infants during RSV season. The American Academy of Pediatrics has issued more clear recommendations for palivizumab use, stating that it should be administered as a monthly injection during RSV season in children born less than 29 weeks, 0 days gestation and are less than 12 months of age or in children with congenital heart disease, chronic lung disease (132). Studies have shown variable efficacy of palivizumab, with reduction in RSV hospitalization rate by approximately 60% (133). Currently, aerosolized ribavirin is the only FDA-approved treatment available for the management of RSV infection, though its use remains controversial (134). To date, RSV vaccines and antiviral therapies remain an active area of investigation (135). A randomized, controlled trial performed in adult patients with RSV infection compared the RSV entry inhibitor GS-5806 to placebo and demonstrated a decrease in both viral load and the clinical severity of infection in patients treated with GS-5806 (136). Similar fusion inhibitors such as ALX-0171 (137), JNJ-2408068 (138), MDT-637 (139), and VP14637 (138) demonstrate efficacy *in vitro*, and ALX-0171 is undergoing a phase II clinical trial in infants hospitalized for RSV (clinicaltrials.gov registration no. NCT02979431). The use of ALS-008176, an RSV polymerase inhibitor, has similarly been shown to reduce viral load, rapidly clear RSV, and improve the severity of disease in adults with RSV infection (140). ALN-RSV01 is a lipid-based nanoparticulate system, containing small-interfering RNA (siRNA] that demonstrates promising antiviral effects against RSV in lung transplant patients (141) by targeting the mRNA of the RSV nucleocapsid protein, thereby limiting viral replication (142). However, until these novel treatments have undergone appropriate clinical trials, the pediatric medical community must continue to wait for effective RSV antiviral therapy.

Unlike RSV, seasonal vaccines and several antiviral therapies are available to treat influenza viral infections. The seasonal influenza vaccine has demonstrated reasonable efficacy at attenuating influenza A and B viral disease (143). Currently, two forms of the influenza vaccine are available for use in children: a live attenuated vaccine in the form of a nasal spray and an inactivated vaccine in an injectable form. Antiviral agents used in the treatment and post-exposure prophylaxis of influenza infections include neuraminidase inhibitors (oseltamivir and zanamivir) and the adamantanes (amantadine and rimantadine). Oseltamivir is the most commonly used medication due to high prevalence of adamantane resistance. Oseltamivir has shown to be beneficial and tolerable in children with influenza if received within first 48 h of illness (144, 145). However, in cases of severe infection, initiation of oseltamivir beyond 48 h of symptom onset may still provide benefit (146). Nanotechnology-based vaccines are also being developed for influenza virus. InflexalR V and InfluvacR are two virosomal vaccines that have been shown to be efficacious against influenza infection (147, 148). STP702, another nanotherapeutic agent, is an siRNA under development designed to inhibit conserved regions in H1N1 and H5N1 strains of the influenza virus and prevent viral replication (116). Nanotraps such as sialylneolacto-N-tetraose c (LSTc)-bearing liposomal decoys bind to hemagglutinins on the influenza virus and prevent viral spread *in* vitro, demonstrating the potential have shown to be effective against influenza virus (117). The influenza polymerase inhibitor T-705 (favipiravir) has been demonstrated significant attenuation of influenza virus activity (149). Interestingly, at higher concentration, it has also shown to be effective against poliovirus, rhinovirus and RSV (149). Other agents under investigation include CS-8958, a long-acting neuraminidase inhibitor, and DAS181, an attachment inhibitor (150). Animal studies have also shown promising results with combination therapy (150). Various immunomodulatory agents have also been posited to temper the dysregulated host inflammatory response in severe influenza (151), including cyclooxygenase-2 inhibitors (152), doxycycline (153), glucocorticoids (154), macrolides (155, 156), peroxisome proliferator-activated receptor agonists such as gemfibrozil (157), sphingosine-1-phosphate (158), and the Tie2 receptor activator vasculotide (65). Further studies are needed to determine the efficacy of these treatments in human influenza infection.

Pleconaril, an orally administered viral capsid inhibitor, has shown to be effective against picornaviruses, especially enteroviruses and rhinoviruses (159). Abzug et al. reported greater survival in patients with neonatal enteroviral sepsis who received pleconaril (159). Similarly, patient with rhinovirus infection treated with pleconaril have shorter duration of symptoms, depending on susceptibility of the virus to the medication (160). No antiviral activity has been observed from pleconaril against HPeV (18). Intravenous immunoglobulin has shown potential benefit in management of enteroviral infections (161).

Prevention of neonatal HSV infection is more elusive as neonatal HSV disease often occurs after transmission from asymptomatic women with primary HSV infection (162). In cases of active maternal genital herpes, cesarean sections can decrease the incidence of neonatal HSV infection, especially when performed within 4 h of rupture of membranes (163). A subunit HSV vaccine has shown promising results in prevention of genital herpes and is currently under Phase III trial (164). Although not routinely recommended, antiviral prophylaxis with acyclovir in late pregnancy has been demonstrated to decrease viral shedding, leading to reduction in cesarean rates and recurrent herpes (165, 166). Patients with severe neonatal HSV infection (those with disseminated disease and CNS infection) should be treated with intravenous acyclovir for 21 days (167).

Viral sepsis may occur in HIV-infected children due to opportunistic or other secondary viral infections (19). Increasing use of highly active antiretroviral therapy (HAART) has significantly improved survival of HIV infected children by decreasing the progression to acquired immunodeficiency syndrome (AIDS), thereby maintaining host immunocompetence that protects against the development of viral sepsis (168–171). However, HAART is associated with potentially deleterious sequelae, making timing of the therapy very controversial in patients with active sepsis (19).

### Outcomes

Extensive studies have not been done to characterize the effect of viral sepsis on outcomes. In a recent study, Hon et al. found no difference in mortality between patients with and without viral infections who were admitted to PICU (172). Shi et al performed a systematic review of RSV infections in 2015 and estimated case fatality rates in children with RSV infection to be around 2.2% (<6 months of age) and 2.4% (6–11 months of age) in developing countries. Case fatality rates in higher income countries were significantly lower (0.2 for <6 months and 0.9 for 6–11 months) (173). In another study, the highest mortality from RSV infection was seen at mean age of 6.2–7.5 months with three quarters of these cases associated with comorbid conditions (174).

Seasonal influenza epidemics and various pandemics have historically led to significant morbidity and mortality in the past, either due to exacerbation of an underlying condition or due to secondary bacterial infections. Mortality with influenza varies not only with season, but with predominant influenza strain and effectiveness of influenza vaccine each season. During the first year of the pandemic 2009 H1N1, global mortality in children aged 0–17 years was estimated to be as high as ~ 45,000 cases, with majority of deaths occurring in Southeast Asia and Africa (175). Both pediatric and adult patients during this pandemic had a very rapid progression to respiratory failure and required prolonged mechanical ventilation and vasopressor support (176, 177). Various extrapulmonary complications secondary to influenza sepsis have been reported in the literature. These include, but are not limited to renal failure, rhabdomyolysis, encephalopathy, myocarditis, and multiorgan failure. These complications also lead to poorer outcomes (178).

Sepsis from HPeV can lead to significant morbidity in neonates and young children. Although, most infections are self-limited, long-term neurological deficits such as learning disability, developmental delay, paralysis and epilepsy have been observed in these patients (179, 180). HPeV infections have also been associated with encephalitis, hepatitis and coagulopathy (18). In addition, rare complications have also been observed in these patients including necrotizing enterocolitis, myocarditis, myositis, hemolytic uremic syndrome, and Reye's syndrome (18). Other enteroviral infections can lead to similar complications and long-term neurological deficits. Hepatic and cardiac dysfunction can also be observed in these patients (181–183). In HSV infection, neurological complications such as developmental delay and seizures have been observed in infected neonates (15). Mortality from systemic HSV infection is usually due to severe coagulopathy, hepatitis and pneumonitis (15). In a multicenter study, Spaeder et al. observed a mortality rate of 9% in patients with severe metapneumovirus infection (184). Increased mortality from metapneumovirus infection has been observed in children with chronic medical conditions, female gender and patients who acquired the infection in the hospital (184). Similarly, RSV, parainfluenza and influenza infections, when acquired in hospital, have been associated with increased mortality (185). Rotavirus infection can lead to extraintestinal complications like seizures and meningoencephalitis (186, 187). In HIV patients, likelihood of progression to AIDS and of mortality are impacted by time of acquisition of HIV, viral load, CD4 count and timing of HAART initiation. Approximately 80% mortality has been observed in developing countries with limited access to HAART (188). Complications that lead to increased morbidity and mortality in these patients include severe CMV infection, encephalopathy, recurrent life-threatening bacterial infections, tuberculosis, and pneumocystis infection (189).

End-organ failure is a major contributor to mortality in sepsis and septic shock, including virus-induced sepsis. Complications such as acute respiratory distress syndrome, disseminated intravascular coagulation, and acute renal injury often leads to a worse prognosis. Developing countries often have disproportionately higher mortality in patients with viral infections (190), likely due to delayed diagnosis and treatment. Risk of severe sepsis is also related to the site of infection, with endocarditis and CNS infections being associated with mortality as high as 20% (1). Besides the site of infection, the type of virus also determines the risk of mortality. For example, meningitis from HPeVs is a common cause of sepsis in neonates and young children but consequent mortality is low in these patients (179). HPeV3 is associated with more severe disease than HPeV1 (113). Moreover, the extent of systemic involvement can predict the development of multiple organ failure and thus mortality (191). Sepsis related mortality has been reported in other viral infections including dengue fever (192). However, further studies are necessary in order to estimate the burden of viral sepsis on outcomes including morbidity, mortality, and health care related costs.

## Summary

Although the incidence of viral-induced sepsis is not precisely known, it is suspected to be common and may represent an important subset of children with “culture-negative sepsis.” It is therefore critical for clinicians to suspect and test for viral infection in children with culture-negative sepsis if appropriate infection containment measures are to be instituted in a timely fashion and in the interest of early identification of children with viral infections amenable to treatment. These considerations are especially urgent for high-risk children, such as those born prematurely or those having congenital heart disease, chronic lung disease, or immunodeficiency. Appropriate diagnosis of viral sepsis may provide the clinician added confidence to limit the duration of empiric antibacterial exposure in children with sepsis, and therefore may be helpful in the fight against antibiotic-resistant bacteria. Further studies are needed to identify novel viral-specific biomarkers and therapeutics.

## Author contributions

NG, RR, SR, and MK contributed to the conception, writing, and final edits of this manuscript.

### Conflict of interest statement

The authors declare that the research was conducted in the absence of any commercial or financial relationships that could be construed as a potential conflict of interest.
